# A population-based survey of the management of women with cancer of the cervix

**DOI:** 10.1038/sj.bjc.6690626

**Published:** 1999-08

**Authors:** F Clarke, P Dey, S Collins

**Affiliations:** Formerly Royal Bolton Hospitals, Minerva Road, Farnworth, UK; Centre for Cancer Epidemiology, University of Manchester, Kinnaird Road, Manchester, M20 4QL, UK

**Keywords:** cancer of the cervix, survival, population-based audit, volume of work, organization of services

## Abstract

The aim of this study was to investigate the influence of diagnostic throughput on survival outcome for women with cancer of the cervix. We conducted a case note review of 359 women in Lancashire and Greater Manchester diagnosed with cancer of the cervix during 1990, identified from records held by the North Western Regional Cancer Registry. Univariate and multivariate survival analyses were undertaken to investigate the influence on survival of woman, disease and treatment related factors. Following adjustment for woman- and disease-related factors there was no evidence of a statistically significant association between diagnostic throughput and survival. The findings of this study do not support the need for any change in the referral patterns to gynaecologists of women with symptoms suggestive of cancer of the cervix. © 1999 Cancer Research Campaign


*British Journal of Cancer* (1999) **80**(12), 1958–1961
© 1999 Cancer Research Campaign
Article no. bjoc.1999.0626

### A population-based survey of the management of women with cancer of the cervix

**F Clarke[1], P Dey[2] and S Collins[2]**

[1]Formerly Royal Bolton Hospitals, Minerva Road, Farnworth, UK. [2]Centre for Cancer Epidemiology, University of Manchester, Kinnaird Road, Manchester M20 4QL, UK

**Summary** The aim of this study was to investigate the influence of diagnostic throughput on survival outcome for women with cancer of the cervix. We conducted a case note review of 359 women in Lancashire and Greater Manchester diagnosed with cancer of the cervix during 1990, identified from records held by the North Western Regional Cancer Registry. Univariate and multivariate survival analyses were undertaken to investigate the influence on survival of woman, disease and treatment related factors. Following adjustment for woman- and disease-related factors there was no evidence of a statistically significant association between diagnostic throughput and survival. The findings of this study do not support the need for any change in the referral patterns to gynaecologists of women with symptoms suggestive of cancer of the cervix.

**Keywords:** cancer of the cervix; survival; population-based audit; volume of work; organization of services

The response by a Joint Working Group of the Royal College of Obstetricians and Gynaecologists and the British Society of Gynaecological Cancer (1997) to the Report of the Expert Advisory Group to the Chief Medical Officers of England and Wales ‘A Policy Framework for Commissioning Cancer Services’ (1995) recommends that women with gynaecological malignancy should be managed by multidisciplinary teams with a minimum acceptable workload. However, while surveys have demonstrated a positive association between throughput and outcome for breast cancer and teratomas (Harding et al, 1993; Sainsbury et al, 1995), this association has not yet been shown for gynaecological malignancies. A recent survey of the management of women with ovarian cancer failed to demonstrate any impact on survival of operator throughput, although it did show a survival advantage for women referred to an oncologist (Woodman et al, 1997). We examine the influence of diagnostic throughput on survival in a population-based series of women with cancer of the cervix.

### MATERIALS AND METHODS

All women resident in Lancashire and Greater Manchester diagnosed with invasive cancer of the cervix (International Classification of Disease 9th revision, code 180) between 1 January 1990 and 31 December 1990 were identified from the database held by the North Western Regional Cancer Registry (NWRCR).

A case note review of these women was undertaken and details on the following variables abstracted from the hospital records: symptoms at presentation; stage; tumour type and grade; consultant specialty; treatment modalities and referral to a clinical oncologist. Stage at time of diagnosis was assessed by one researcher (FC) using FIGO classification (1987).

Survival time was measured from date of first treatment or, if not treated, date first seen, until date of death from any cause and was censored after 31 December 1995. Vital status was ascertained from records held by the NWRCR. Univariate survival estimates were calculated using the Kaplan–Meier method and compared using log-rank tests of significance (Kaplan and Meier, 1958). Multivariate analyses were undertaken using Cox’s proportional hazards model to investigate, simultaneously, the influence on survival of patient- and disease-related factors (Cox, 1972).

Clinicians responsible for the diagnosis of the women in this series were categorized as either gynaecologists or non-gynaecologists, but investigation of the association between diagnostic throughput and outcome was restricted to gynaecologists. The distribution of throughput values was divided into two or more categories of contiguous values in as many ways as possible, producing an exhaustive set of variables. Those variables for which any category contained fewer than 10% of the study population were excluded. The remaining throughput variables were then examined in univariate and multivariate analyses.

### RESULTS

A total of 381 eligible women were diagnosed with cancer of the cervix during the study period. Excluded from further analysis were seven women who were registered solely from death certificates, and 15 women for whom case notes were not available.

The characteristics of the remaining 359 (94%) women are shown in Table 1. The mean age of these women was 53 years. Stage was assigned by the author (FC) for 349 (97%) women, although it was only explicitly documented in the case notes of 183 (51%) women. The stage assigned by the author corresponded with that recorded by the clinician in 171 (93%) cases.

Univariate analyses identified age, stage, histology, presenting symptom, place of diagnosis and referral to clinical oncologist

*Received 16 April 1998*
*Revised 15 January 1999*
*Accepted 20 February 1999*

*Correspondence to:* P Dey

**Table 1** Distribution of population characteristics and univariate survival analysis of 359 women with cancer of the cervix

| | | | Survival % | | |
|---|---|---|---|---|---|
| | ***n*** | **%** | **1-year** | **3-year** | **5-year** |
| **All cases** | **359** | **100.0** | **82.73** | **67.41** | **61.00** |
| Age | | | | | |
| [$\chi^2_{(4)}$ = 57.85, $P < 0.001$][a] | | | | | |
| 0–39 | 87 | 24.2 | 95.40 | 86.21 | 85.06 |
| 40–49 | 80 | 22.3 | 90.00 | 80.00 | 71.25 |
| 50–59 | 64 | 17.8 | 85.94 | 60.94 | 54.69 |
| 60–69 | 66 | 18.4 | 71.21 | 56.06 | 46.97 |
| 70+ | 62 | 17.3 | 64.52 | 43.55 | 35.48 |
| Differentiation | | | | | |
| [$\chi^2_{(3)}$ = 2.21, $P = 0.53$][a] | | | | | |
| Well | 40 | 11.1 | 85.00 | 75.00 | 67.50 |
| Moderate | 86 | 24.0 | 77.91 | 63.95 | 56.98 |
| Poor | 111 | 30.9 | 84.68 | 66.67 | 59.46 |
| Unknown | 122 | 34.0 | 83.61 | 68.03 | 63.11 |
| Stage | | | | | |
| [$\chi^2_{(4)}$ = 230.81, $P < 0.001$][a] | | | | | |
| I | 207 | 57.7 | 96.62 | 87.44 | 83.09 |
| II | 79 | 22.0 | 79.75 | 53.16 | 37.97 |
| III | 46 | 12.8 | 52.17 | 28.26 | 26.09 |
| IV | 17 | 4.7 | 23.53 | 5.88 | 0.00 |
| Unknown | 10 | 2.8 | 60.00 | 50.00 | 50.00 |
| Histology | | | | | |
| [$\chi^2_{(2)}$ = 13.64, $P < 0.01$][a] | | | | | |
| Squamous+adenosquamous | 300 | 83.6 | 85.33 | 69.33 | 62.67 |
| Adenocarcinoma | 36 | 10.0 | 80.56 | 69.44 | 63.89 |
| Others | 23 | 6.4 | 52.17 | 39.13 | 34.78 |
| Symptom | | | | | |
| [$\chi^2_{(2)}$ = 133.53, $P < 0.001$][a] | | | | | |
| PV bleed | 199 | 55.4 | 81.91 | 62.31 | 52.26 |
| Abnormal smear | 122 | 34.0 | 98.36 | 90.98 | 89.34 |
| Others | 38 | 10.6 | 36.84 | 18.42 | 15.79 |
| Place of diagnosis | | | | | |
| [$\chi^2_{(2)}$ = 69.88, $P < 0.001$][a] | | | | | |
| Outpatient | 238 | 66.3 | 79.83 | 62.18 | 54.20 |
| Colposcopy | 102 | 28.4 | 97.06 | 89.22 | 85.29 |
| Inpatient | 19 | 5.3 | 42.11 | 15.79 | 15.79 |
| Referral to oncologist | | | | | |
| [$\chi^2_{(1)}$ = 16.81, $P < 0.001$][a] | | | | | |
| Not referred | 115 | 32.0 | 82.61 | 79.13 | 78.26 |
| Referred | 244 | 68.0 | 82.79 | 61.89 | 52.87 |

[a]Log-rank test for heterogeneity.

as statistically significant prognostic variables (Table 1). In a multivariate analysis, age, stage, histology and presenting symptom remained statistically significant independent prognostic variables (Table 2). Adverse prognostic factors were late stage at presentation, uncommon histology and presentation with non-gynaecological symptoms. Women referred to a clinical oncologist had better outcome than those not referred but the observed difference was not statistically significant ($\chi^2$ = 3.40, df = 1, $P$ = 0.07) (Table 2).

Women in this series were referred to 33 provider units; 23 (6%) women were initially referred to non-gynaecologists, of whom 12 were subsequently transferred to the care of a gynaecologist. Of the 348 woman assessed by a consultant gynaecologist, 97 (28%) were assessed by gynaecologists diagnosing five or fewer cases in 1990, 153 (44%) by gynaecologists diagnosing between six and ten cases and 97 (28%) by gynaecologists diagnosing more than ten cases; the identity of the gynaecologist could not be determined for one woman. No consistent evidence of an association between the diagnostic throughput of the gynaecologist and survival outcome was found either in the univariate or multivariate analyses; this was true regardless of how the diagnostic workload variable was defined. Relative risks of death (hazards ratios) for a selection of workload variables are presented in Table 3.

Of the 182 (52%) women with stage IB/IIA cancer of the cervix, 97 (53%) were treated primarily with radiotherapy; 66 (36%) by radical hysterectomy and two women refused treatment. For the remaining 17 (9%) women, cancer had been an unexpected finding at the time of a total abdominal hysterectomy which had been undertaken for other reasons: ten women had had a preoperative diagnosis of cervical intraepithelial neoplasia made at colposcopy; three women had undergone hysterectomy for dysfunctional uterine bleeding without a preceding cervical smear and the remaining four women had presented with an abnormal smear but had not undergone preoperative colposcopy. This failure to

*© 1999 Cancer Research Campaign*

**Table 2** Determinants of outcome: multivariate survival comparison

| | Number of deaths prior to 1 January 1996 | Relative risk (ratio of hazards) | 95% Confidence interval |
|---|---|---|---|
| Age | | | |
| 0–39 | 13 | 1.00 | – Baseline – |
| 40–49 | 24 | 1.59 | 0.79 to 3.18 |
| 50–59 | 29 | 2.85 | 1.44 to 5.61 |
| 60–69 | 35 | 2.43 | 1.23 to 4.79 |
| 70+ | 42 | 2.70 | 1.39 to 5.22 |
| | | | $\chi^2_{(4)}$ = 12.92, $P$ < 0.05[a] |
| Stage | | | |
| I and not known | 42 | 1.00 | – Baseline – |
| II | 50 | 3.67 | 2.28 to 5.90 |
| III + IV | 51 | 5.21 | 3.17 to 8.58 |
| | | | $\chi^2_{(2)}$ = 45.31, $P$ < 0.0001[a] |
| Degree of differentiation | | | |
| Well | 13 | 1.00 | – Baseline – |
| Moderate | 39 | 1.02 | 0.54 to 1.94 |
| Poor | 46 | 1.24 | 0.66 to 2.34 |
| Not known | 45 | 0.80 | 0.42 to 1.55 |
| | | | $\chi^2_{(3)}$ = 3.68, $P$ = 0.30[a] |
| Histological type | | | |
| Squamous & adenosquamous | 115 | 1.00 | – Baseline – |
| Adenocarcinoma | 13 | 1.24 | 0.69 to 2.24 |
| Others | 15 | 2.84 | 1.52 to 5.29 |
| | | | $\chi^2_{(2)}$ = 11.06, $P$ < 0.01[a] |
| Presenting symptom | | | |
| PV bleed | 98 | 1.00 | – Baseline – |
| Abnormal smear | 13 | 0.33 | 0.17 to 0.63 |
| Others | 32 | 3.66 | 2.33 to 5.77 |
| | | | $\chi^2_{(2)}$ = 51.02, $P$ < 0.0001[a] |
| Oncologist | | | |
| Not referred | 25 | 1.00 | – Baseline– |
| Referred | 118 | 0.61 | 0.36 to 1.03 |
| | | | $\chi^2_{(1)}$ = 3.40, $P$ = 0.07[a] |

[a]Wald test for heterogeneity adequately assess women prior to surgery could not be related to diagnostic throughput: four (24%) women were assessed by gynaecologists with a diagnostic throughput of five or fewer cases; six (35%) by gynaecologists with a diagnostic throughput of between six and ten cases; and seven (41%) by gynaecologists diagnosing more than ten cases ($\chi^2$ = 1.89, df = 2, $P$ = 0.39).

### DISCUSSION

This analysis failed to demonstrate an association between diagnostic throughput and survival. The low rate of operative intervention in this series of women precluded any further examination of the impact of operator throughput on survival.

In order to demonstrate an association between throughput and survival it is first necessary to reveal heterogeneity in the uptake of an intervention of proven efficacy and then to further demonstrate an association between heterogeneity of uptake of this intervention and institutional or clinical throughput (Sainsbury et al, 1995). When the uptake of effective interventions is dependent on the adequacy of the initial assessment and this varies between clinicians, then it would seem reasonable to further investigate the possibility of a relationship between diagnostic throughput and survival.

Audits of the management of women with cervical cancer have demonstrated variation between institutions in the frequency with which certain investigations are undertaken (Wolfe et al, 1996; Jackson et al, 1997). Our survey also revealed suboptimal diagnostic practice. Only two-thirds of pathology reports contained a comment on tumour grade but neither univariate nor multivariate analysis confirmed tumour grade as an important prognostic variable. In addition, 17 women received inadequate surgery following a failure to exclude the presence of cancer of the cervix at initial assessment. However, further analysis failed to demonstrate an association between inadequate initial assessment and diagnostic throughput.

Arguably, the benefits that might accrue from the centralization of gynaecological cancer services are not solely measurable in terms of survival outcome. Multidisciplinary specialist care might also be expected to reduce morbidity and increase patient satisfaction. However, little work has been undertaken to validate other measures of the quality of gynaecological cancer services.

The Joint Working Party recommends that women with gynaecological malignancy are managed in fewer units (Joint Working Group, 1997). Multidisciplinary teams in gynaecological oncology centres would manage women who require radiotherapy, chemotherapy or specialist surgery, and multidisciplinary teams in associate units would manage women requiring less specialized surgery. Apart from central pathological review, there are no specific recommendations on who might best initially assess women with symptoms suggestive of gynaecological cancer, but

 *© 1999 Cancer Research Campaign*

**Table 3** Relative hazard ratios[a] for varying combinations of diagnostic throughput of individual gynaecologists. (Each row in the table refers to a different diagnostic throughput variable. For any given row, the range of throughput to which the hazards ratio applies is determined by projecting the vertical lines which separate the ratios to meet the distribution of throughput values at the top of the table. The reference (baseline) group is shaded)

[a]Relative hazard ratios for different combinations of throughput, derived from a proportional hazards model controlling for age, degree of differentiation, stage, histology, presenting symptom and referral to clinical oncologist. [b]Twelve women were excluded from this analysis: ten women were not diagnosed by a gynaecologist; the identity of the diagnosing gynaecologist was unknown for a further two women. [c]Nominal *P*-value only. Several hundred hypotheses regarding diagnostic throughput were tested on this data, so the true *P*-value would need adjusting to account for multiple testing.

many gynaecologists anticipate a shift in primary care referrals from ‘non designated’ units to multidisciplinary teams in associate units or gynaecological oncology centres. Cancer of the cervix most frequently presents following an abnormal smear or vaginal bleeding; any major shift in the referral of women with these symptoms to cancer centres or associate units could compromise the viability of general gynaecological services in ‘non designated’ units. If the benefits of centralization are measured solely by survival, then the findings of this study do not support any change to the current pattern of referral of women with symptoms suggestive of cancer of the cervix to general gynaecologists. Further work is required to determine if these conclusions can be confirmed for other common gynaecological malignancies.

### ACKNOWLEDGEMENTS

FC was a Joseph Starkey Fellow, Department of Immunology, Christie CRC Research Centre, Paterson Institute for Cancer Research during this study. We would like to thank Dr Peter Stern and the Department of Immunology for support and discussion during this study and Professor Ciaran Woodman, Director, Centre for Cancer Epidemiology for his advice and support.

### REFERENCES

Cox DR (1972) Regression models of life tables. *J R Stat Soc* **34**: 187–220

Expert Advisory Group on Cancer (1995) *A Policy Framework for Commissioning Cancer Services (Report to the Chief Medical Officer of England and Wales)*. HMSO: London

FIGO News (1987) *Int J Gynaecol Obstet* **25**: 87

Harding MT, Paul J, Gillis CR and Kaye SB (1993) Management of malignant teratoma: does referral to a specialist unit matter? *Lancet* **341**: 999–1002

Jackson S, Murdoch J, Howe K, Bedford C, Sanders T and Prentice A (1997) The management of cervical carcinoma within the south west of England. *Br J Obstet Gynaecol* **104**: 140–144

Joint Working Group Response by The Royal College of Obstetricians and Gynaecologists and The British Gynaecological Cancer Society (1997) *A Policy Framework for Commissioning Cancer Services*. RCOG Press: London

Kaplan EL and Meier P (1958) Non-parametric estimation from incomplete observations. *J Am Stat Assoc* **53**: 457–481

Sainsbury R, Howard B, Rider L, et al. (1995) Influence of clinical workload and patterns of treatment on survival from breast cancer. *Lancet* **345**: 1265–1270

WHO (1977) *Manual of the International Statistical Classification of Diseases, Injuries and Causes of Death*, 9th Revision. World Health Organization: Geneva

Wolfe CA, Tilling K, Bourne HM and Raju KS (1996) Variations in the screening history and appropriateness of management of cervical cancer in south east England. *Eur J Cancer* **32A**: 1198–1204

Woodman C, Baghdady A, Collins S and Clyma JA (1997) What changes in the organisation of cancer services will improve the outcome for women with ovarian cancer? *Br J Obstet Gynaecol* **104**: 135–139

© 1999 Cancer Research Campaign 
